# Supplementary material for: Antifungal Activity of Cedrol from Cunninghamia lanceolate var. konishii against Phellinus noxius and Its Mechanism
Source: Plants (Basel). 2024 Jan 21;13(2):321. doi: 10.3390/plants13020321 (PMC10821468; doi:10.3390/plants13020321)
Supplement: Supplementary file 1 [file plants-13-00321-s001.zip › plants-2825636-supplementary.pdf]

Supporting Information

Antifungal Activity of Cedrol from *Cunninghamia lanceolate* var. *konishii* against *Phellinus noxius* and Its Mechanism

Wen-Wei Hsiao [1], Ka-Man Lau [2], Shih-Chang Chien [3], Fang-Hua Chu [4], Wen-Hsin Chung [5] and Sheng-Yang Wang [2,6,7,*]

[1] Experimental Forest, College of Bio-Resources and Agriculture, National Taiwan University, Taipei 10617, Taiwan; hsiaoww@gmail.com
[2] Department of Forestry, National Chung Hsing University, Taichung 40202, Taiwan; kakablauu@gmail.com
[3] Experimental Forest Management Office, National Chung Hsing University, Taichung 40202, Taiwan; scchien@dragon.nchu.edu.tw
[4] School of Forestry and Resource Conservation, National Taiwan University, Taipei 106217, Taiwan; fhchu@ntu.edu.tw
[5] Department of Plant Pathology, National Chung Hsing University, Taichung 40202, Taiwan; wenchung@nchu.edu.tw
[6] Special Crop and Metabolome Discipline Cluster, Academy Circle Economy, National Chung Hsing University, Taichung *40202*, Taiwan
[7] Agricultural Biotechnology Research Center, Academia Sinica, Taipei 11529, Taiwan
* Correspondence: taiwanfir@dragon.nchu.edu.tw; Tel.: +886-422850333; Fax: +886-422862960

Table S1. SDS-PAGE formula.

Chemical components	Separating gel				Stacking gel
	8%	10%	12%	15%	
ddH_2O	4600	4000	3300	2300	1700
1.5M Tris-HCl	2500	2500	2500	2500	-
0.5M Tris-HCl	-	-	-	-	750
30% Acrylamide/Bis	2700	3300	4000	5000	500
10% SDS	100	100	100	100	30
10% Ammonium persulfate	100	100	100	100	30
TEMED	6	4	4	4	3

Unit: μL, microliter

Table S2. Antibody products list.

Antibodies	Manufacturers	#
GAPDH	Arigo Biolaboratories	ARG62345
Cytochrome c	Cell Signaling Technology	11940
Caspase-9	Cell Signaling Technology	9502
Caspase-3	Cell Signaling Technology	9662
PARP	Cell Signaling Technology	9542
Apaf-1	Cell Signaling Technology	8969
AIF	Cell Signaling Technology	4642
